# Transcutaneous spinal direct current stimulation shows no effect on paired stimulation suppression of the somatosensory cortex

**DOI:** 10.1038/s41598-020-79131-2

**Published:** 2020-12-15

**Authors:** Jan H. Bettmann, Christine H. Meyer-Frießem, Lauren M. Schweizer, Lara Schlaffke, Peter K. Zahn, Martin Tegenthoff, Oliver Höffken

**Affiliations:** 1 Department of Neurology, BG-Universitätsklinikum Bergmannsheil gGmbH, Bürkle-de-la-Camp-Platz 1, 44789 Bochum, Germany; 2 Department of Anesthesiology, Intensive Care and Pain Management, BG-Universitätsklinikum Bergmannsheil gGmbH, Bürkle-de-la-Camp-Platz 1, 44789 Bochum, Germany; 3 Present Address: Department of Trauma Surgery, Orthopedic Surgery and Hand Surgery, Agaplesion Bethesda Krankenhaus Wuppertal gGmbH, Hainstraße 35, 42109 Wuppertal, Germany

**Keywords:** Neuroscience, Neurology

## Abstract

Transcutaneous spinal direct current stimulation (tsDCS) is a safe and convenient method of neuromodulation. It has been proven to alter sensory processing at cervicomedullary level by amplitude changes of the P30 response of tibial nerve somatosensory evoked potentials (TN SEPs). With knowledge that tsDCS affects cortical circuits, we hypothesized that tsDCS may also affect intracortical excitability of the somatosensory cortex assessed by paired stimulation suppression (PSS). Fourteen healthy men were included in this prospective, single-blinded, placebo-controlled crossover study. Single (SS) and paired stimulation (PS) TN SEPs were recorded over the scalp before, immediately as well as 30 and 60 min after applying 15 min of tsDCS over the twelfth thoracic vertebra. Each volunteer underwent three independent and randomized sessions of either cathodal, anodal or sham stimulation. tsDCS showed no effect on peak-to-peak amplitudes or latencies of cortical P40-N50 response after SS. Furthermore, tsDCS failed to induce significant changes on amplitude ratios of PSS, thus showing no impact on intracortical excitability of the somatosensory cortex in healthy subjects. Further research is required to reveal the different mechanisms and to strengthen clinical use of this promising technique.

## Introduction

Modulation of cortical excitability and plasticity using non-invasive and weak electric currents to stimulate neural tissue is a recent topic of neuroscience^1–3^. Transcranial direct current stimulation has been shown to induce lasting changes in cortical neural activity^4–7^ and is established as a clinical tool by now^8–10^ resulting in level B evidence for treating fibromyalgia, depression and craving^1^. A similar approach is the application of homologous direct currents to the spinal cord: transcutaneous spinal direct current stimulation (tsDCS).

Invasive pulsed spinal cord stimulation (SCS) induces cortical changes described as alterations of SEP amplitudes or pain perception in humans^11–13^. In animal studies, invasive spinal direct current stimulation showed polarity-specific and state-dependent supraspinal effects on the activity of the gracile nucleus and the primary somatosensory cortex^14^. Analogously, non-invasive tsDCS has been proven to influence ascending pathways including the nociceptive system in humans^15–19^. TsDCS affects descending pathways^20–22^ and spinal reflexes of different levels^23–27^ resulting in first therapeutic approaches in patients with restless legs syndrome^28^, hereditary spastic paraplegia^29^ or spinal cord injuries^24,30,31^. Additionally, tsDCS significantly alters amplitudes of motor evoked potentials after paired-pulse transcranial magnetic stimulation^32^, decreases intracortical inhibition and increases intracortical facilitation^33^ underlining the impact of tsDCS on motor cortex excitability and descending pathways.

By analyzing TN SEPs, previous studies showed that anodal tsDCS of the lower thoracic spine induces subcortical changes in the neural activity of the brainstem represented by an amplitude depression of the far field potential P30 recorded over the sixth cervical vertebra while cathodal tsDCS tends to increase P30^15^, both suggesting axonal alterations of transmission. First evidence of tsDCS inducing effects within the somatosensory cortex was recently revealed by illustrating cortical changes of somatosensory functional connectivity in a fMRI study^34^. However, knowledge of tsDCS’ impact on the somatosensory cortex remains rare.

A more specific approach of deducing intracortical excitability changes of the somatosensory cortex compared to the sole analysis of amplitudes is the evaluation of paired stimulation suppression (PSS) patterns of SEPs. PSS is a common tool to examine neuronal excitability and serves as a model for investigating short-term plasticity^35–37^. By assessing the suppressive effect of a stimulus on a subsequent second stimulus PSS is supposed to measure intracortical excitability^35,38,39^. Previous studies demonstrated the cortical origin of PSS by using recording techniques at different hierarchical levels of the sensory pathway^36,40,41^. Based on findings in pharmacological studies, there is strong evidence, that PSS is mainly GABA-mediated^39^. Furthermore, changes in PSS in the somatosensory system as an indicator of facilitatory and inhibitory effects have been studied in several neurological disorders^38,42,43^.

Until now, there is no data available concerning the effect of tsDCS on PSS of the somatosensory cortex. To further understand the underlying mechanisms of tsDCS, this prospective volunteer study was designed with the hypothesis that tsDCS will alter the intracortical excitability of the somatosensory cortex expressed by alterations of PSS following TN SEPs.

## Results

**Figure 1:**
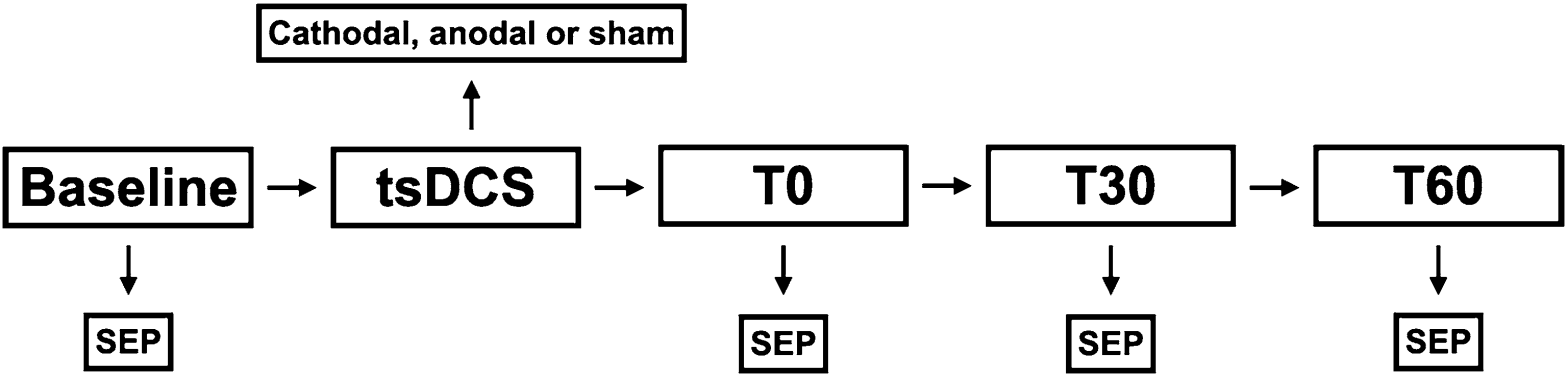
Study design: each study participant underwent three individual sessions of the procedure shown above with a latency of at least 1 week between these sessions. During each session 15 min of either cathodal, anodal or sham transcutaneous spinal direct current stimulation (tsDCS) was applied to the participant. Before tsDCS, a baseline tibial nerve SEP was recorded (Baseline). tsDCS was then applied in randomized individual order. The stimulation was followed by three SEP recordings with the first one being started directly after tsDCS (T0). Another two SEP recordings were performed 30 (T30) and 60 (T60) min after tsDCS.

One out of fifteen participants was excluded from further analysis due to electroencephalogram artefacts. Table 1. Independent from stimulation polarity (Fig. 1), participants did not report any adverse events due to tsDCS. See Supplement 1 for raw data.

**Table 1:** Clinical characteristics of volunteers.

Number	n = 14
Age	24.43 ± 2.53 years
Height	1.78 ± 0.06 m
Body mass index	23.05 ± 2.09 kg/m^2^
Reported adverse events	n = 0
Sensory threshold	7.28 ± 0.19 mA
Motor threshold	9.78 ± 0.23 mA
Applied current	12.27 ± 0.24 mA

Data given in mean ± SD.

### Single stimulation

Baseline SEPs were comparable throughout all sessions (amplitudes: F_2, 39_ = 0.484, p = 0.62; latencies: F_2, 39_ = 0.015, p = 0.985).

**Figure 2:**
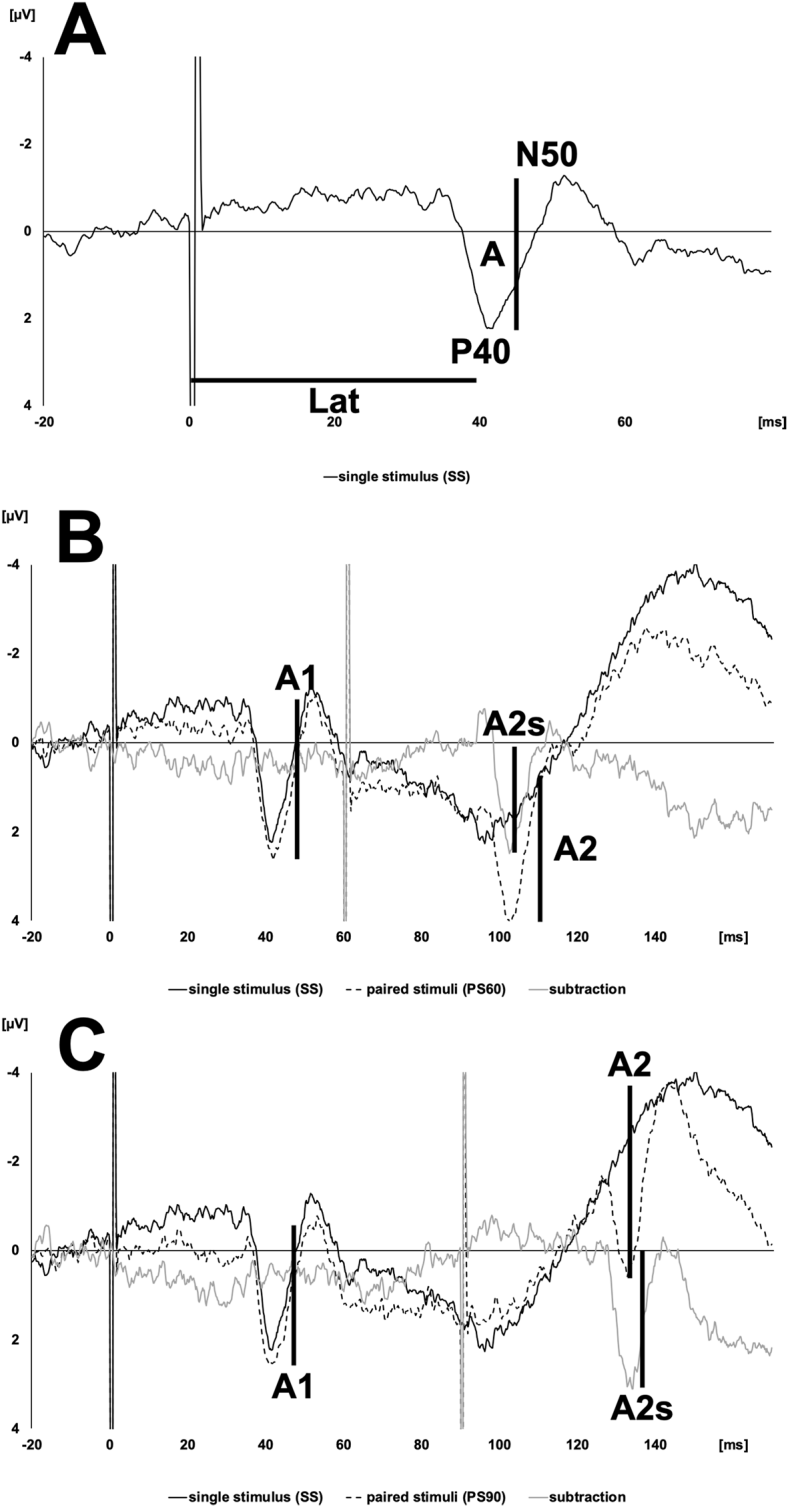
(**A**) Example of a single stimulation tibial nerve somatosensory evoked potential recorded over Cz’. “A” shows the amplitude (µV) and “Lat” the latency (ms) of the P40-N50 peak. (**B**/**C**) Examples of a paired stimulation tibial nerve somatosensory evoked potentials recorded over Cz’ with an interstimulus interval of 60 (PS60; **B**) and 90 (PS90; **C**) ms. “A1” shows the amplitude (P40-N50; µV) of the first peak as “A2” shows the amplitude of the second peak. “A2s” displays the amplitude of the second peak after linear subtraction of the response to a single stimulation. y-axis: amplitude [µV]. x-axis: time [ms].

No main effects of tsDCS were seen in amplitudes (polarity: F_1.88, 15.02_ = 0.329, p = 0.712, partial η^2^ = 0.039; time: F_1.55, 12.37_ = 1.106, p = 0.345, partial η^2^ = 0.121). In latencies only a main effect over time was observed (polarity: F_1.89, 15.15_ = 0.32, p = 0.719, partial η^2^ = 0.038; time: F_2.57, 20.53_ = 5.837, p = 0.006, partial η^2^ = 0.422). Also, no interactions between polarity*time were detected (amplitudes: F_3.16, 25.26_ = 1.378, p = 0.272, partial η^2^ = 0.147; latencies: F_3.33, 26.62_ = 1.416, p = 0.259, partial η^2^ = 0.15). Figure 2A, Table 2. Polarity sequence did not influence the results.

**Table 2:** Amplitudes and latencies of single stimulations.

Polarity	Time	Amplitude of P40-N50 (µV) [mean ± SD]	Amplitude of P40-N50 p-value	Latency of P40-N50 (ms) [mean ± SD]	Latency of P40-N50 p-value
Cathodal tsDCS	Baseline	2.11 ± 0.89	p = 0.272	42.4 ± 2.8	p = 0.259
	T0	2.03 ± 0.88		42.5 ± 2.7	
	T30	1.99 ± 1.09		42.9 ± 2.9	
	T60	2.01 ± 1.15		42.5 ± 2.8	
Anodal tsDCS	Baseline	2.22 ± 0.77		42.3 ± 3.0	
	T0	2.19 ± 0.92		42.7 ± 3.0	
	T30	2.00 ± 0.87		43.0 ± 3.0	
	T60	2.00 ± 0.70		42.6 ± 3.1	
Sham tsDCS	Baseline	1.89 ± 1.04		42.5 ± 2.8	
	T0	1.92 ± 0.89		42.6 ± 2.9	
	T30	2.02 ± 1.03		42.8 ± 2.7	
	T60	1.83 ± 1.09		42.8 ± 2.9	

Amplitudes (µV) and latencies (ms) (mean ± SD) of P40-N50 peak of tibial nerve SEP. SEPs were recorded before (baseline), immediately (T0) as well as 30 (T30) and 60 min (T60) after tsDCS. P-values were calculated using a Greenhouse–Geisser-corrected rmANOVA for the interaction polarity*time; n = 14.

### Paired stimulation (PS60 and PS90)

**Figure 3:**
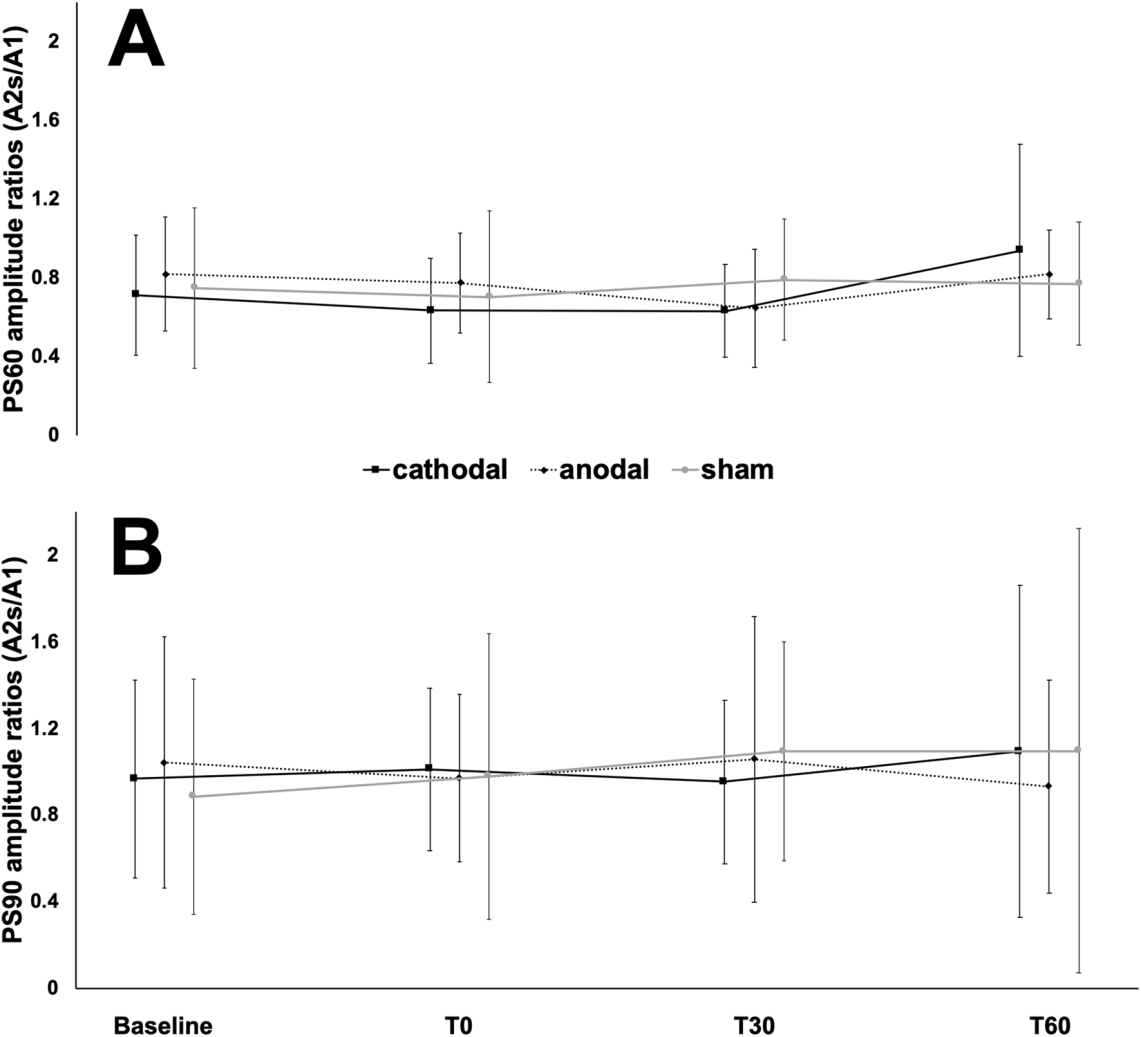
(**A**/**B**) The plots show the progress of paired stimulation suppression ratios (A2s/A1) recorded over Cz’ following stimulation of the tibial nerve with an interstimulus interval of 60 (PS60; **A**) and 90 (PS90; **B**) ms. The black line shows progress after cathodal, the dotted line after anodal and the grey line after sham transcutaneous spinal direct current stimulation. Recordings took place before (baseline), immediately (T0) as well as 30 (T30) and 60 (T60) min after stimulation. y-axis: amplitude ratios (A2s/A1). x-axis: points of time.

An interaction of polarity*time was revealed for PS60 (F_3.54, 28.34_ = 3.857, p = 0.015, partial η^2^ = 0.325), but no main effects on polarity (F_1.84, 14.75_ = 0.268, p = 0.751, partial η^2^ = 0.032) or time (F_2.25, 18.02_ = 3.208, p = 0.059, partial η^2^ = 0.286). Figures 2B and 3A, Table 3. Focusing on PS90, no interaction between polarity*time could be explored (F_3.07, 24.6_ = 2.86, p = 0.056, partial η^2^ = 0.263). Also, there were no effects of tsDCS polarity (F_1.81, 14.48_ = 0.886, p = 0.424, partial η^2^ = 0.1) or over time (F_1.4, 11.21_ = 4.021, p = 0.06, partial η^2^ = 0.334). Figures 2C and 3B, Table 3. Polarity sequence did not influence the results.

**Table 3:** Amplitude ratios of paired stimulations.

Polarity	Time	PS60 A2s/A1 [mean ± SD]	PS60 p-value	PS90 A2s/A1 [mean ± SD]	PS90 p-value
Cathodal tsDCS	Baseline	0.71 ± 0.31	p = 0.015	0.97 ± 0.46	p = 0.056
	T0	0.63 ± 0.27		1.01 ± 0.37	
	T30	0.63 ± 0.24		0.95 ± 0.38	
	T60	0.94 ± 0.54		1.09 ± 0.77	
Anodal tsDCS	Baseline	0.82 ± 0.29		1.04 ± 0.58	
	T0	0.77 ± 0.25		0.97 ± 0.39	
	T30	0.65 ± 0.30		1.06 ± 0.66	
	T60	0.82 ± 0.23		0.93 ± 0.49	
Sham tsDCS	Baseline	0.75 ± 0.41		0.89 ± 0.54	
	T0	0.70 ± 0.44		0.98 ± 0.66	
	T30	0.79 ± 0.31		1.09 ± 0.51	
	T60	0.77 ± 0.31		1.10 ± 1.03	

Ratios of the P40-N50 peaks (A2s/A1) of paired stimulations with an interstimulus interval of 60 (PS60) and 90 ms (PS90) (mean ± SD). SEPs were recorded before (baseline), immediately (T0) as well as 30 (T30) and 60 min (T60) after tsDCS. P-values were calculated using a Greenhouse–Geisser-corrected rmANOVA for polarity*time; n = 14.

## Discussion

Previously unaddressed, the present study aimed to evaluate cortical modulatory effects of tsDCS in healthy subjects. Against expectations, tsDCS did not alter the intracortical excitability of the primary somatosensory cortex assessed by paired stimulation of TN SEPs.

Even though there is evidence of tsDCS influencing cortical circuits and functional connectivity offside its site of application^15,16,32–34,44^, there is no data available yet on its effect on the intracortical excitability of the primary somatosensory cortex. By evaluating the suppressive effect of a precursory stimulus on a subsequent stimulus of an evoked cortical potential, PSS is a commonly used tool to estimate intracortical excitability^35,36,45–47^ and seems to reflect inhibitory cortical processes^36,48^. Especially presynaptic mechanisms are believed to be essential for PSS^39,49^. In animal trials presynaptic GABA_A_^50^ and GABA_B_^51,52^ receptors have been identified to be involved in PSS, which is dynamic and declines with age in humans^46^.

The missing effect is surprising, as clinically established invasive procedures on the spinal cord such as pulsed SCS have already been proven to affect cortical TN SEP components in terms of amplitude depressions^11^ deducing that pulsed stimulation—may it be invasive or transcutaneous^53^—seems to be one major factor of efficacy. Specifying on transcutaneous SCS over the cervical spinal cord, the carrier frequency is essential for modulating intracortical inhibition of the motor cortex^53^.

Yet, non-invasive anodal tsDCS has been demonstrated to attenuate TN SEP amplitudes on a subcortical level whereas cortical SEP components were unaffected^15^, which is in line with our findings. The authors argued, that the far field potential P30, originated in the cervicomedullary junction^54^, is more sensitive than the near field potential P40-N50, generated within the primary sensory cortex^54–57^, for spatial and temporal synaptic summation may have equalized possible cortical effects^15,58^. However, in our setup, which was designed to be a more specific approach of deducing intracortical excitability by analyzing PSS rather than sole amplitudes, after both cathodal and anodal tsDCS there were no relevant changes in neither amplitudes nor latencies of SS nor ratios of paired stimulation amplitudes of the P40-N50 response over Cz’ up to one hour after tsDCS. Hence, we must conclude that tsDCS does not affect inhibitory cortical processes of the somatosensory cortex.

Supporting our results, recent research showed a selective effect of tsDCS on nociception models which were predominantly forwarded by thinly myelinated Aδ fibers^16–18,25^. In other words, it is likely that thickly myelinated Aβ nerve fibers remain largely unaffected by tsDCS, as seen by SEP transmission. Doing so, tsDCS may lead to quantifiable subcortical changes causing an altered pain perception but leaving the intracortical excitability of the somatosensory cortex unchanged.

Regardless of our findings and the exact mechanisms, tsDCS has been shown to be a promising technique in pathological conditions like pain^16,17,19^, post-stroke-conditions^44^, restless legs syndrome^28^ or spinal cord injuries^24,31^. Those effects of tsDCS rely mainly on pathological or sensitized preconditions also seen in experimental settings for presupposed pain^18^ or in combination e.g. with locomotor learning^59^. Approaches to assess effects of SCS on SEPs also premised pain^11–13^. Additionally, invasive spinal direct current stimulation in rats caused changes in cortical evoked potentials which were dependent on cortical spontaneous activity^14^. The dependency of cortical plasticity on cofactors is renowned^60^ as is the pharmaceutical modulation of PSS on for example the somatosensory^39^ or the visual cortex^61^ depicting hypothetic requirements that were not available in our setup. Concluding that tsDCS predominantly affects specific or pathological preconditions, further research should be conducted especially in patients.

PSS of the TN is not as common as of the median nerve. Literature is not providing standard procedures and parameter settings. Yet this setup was chosen to be comparable with the one of Cogiamanian et al. who earlier demonstrated an effect of tsDCS on SEPs at cervicomedullary level^15^. In addition, there are no universal standards defining the amplitudes of TN SEPs. In order to focus on intracortical excitability, the presented study—contrasting^15^—not only takes the approach of analyzing PSS instead of mere SS amplitudes but also defines the SEP amplitude as peak to peak of P40 to N50 as these potentials are assumed to be generated intracortically^11,55,62,63^. Furthermore, even in accordance with the guidelines of the International Confederation of Clinical Physiology, there is a vast number of possible electrode configurations and recording techniques involving equipment, montages, filters, interfering signals, stimulus rates or the type of stimulation electrodes^64^. Limiting the time of data acquisition for each SEP to 10 min for the repetitive setup of a single stimulation followed by two paired stimulations at 2 Hz would allow the acquisition of 400 cycles which is comparably less considering the possible alterations of the second phase.

Additionally, the sample size of fourteen participants is low, but was based on comparable tsDCS studies, which are typically around 12–15 subjects^15,16,23,25,26^. However, effect sizes underline our results. Single changes found over time without any effect due to tsDCS seem to be inconclusively and should be neglected.

Furthermore, the exact spread of the electric field of tsDCS in the human body is unknown yet as are the mechanisms and the targets of the technique leading to a lack of standardization of electrode characteristics, electrode placement and energy charge during application. Considering the spread of the electric field, body positioning during the application seems to be a crucial factor as a significant increase of motor evoked potentials after cathodal tsDCS has been shown when tsDCS was applied to the proband in supine position whereas in seated position there were no significant effects^65^. Again, stimulation patterns (e.g., pulsed^11,13^ versus continuous DCS, stimulation repetition, duration^34,65^ and intensity) may vary the effects explaining heterogenous results. As SEPs were not recorded during tsDCS, an early effect may have been missed. The latter aspects should be addressed in future research.

In conclusion, against expectations, the current results did not reveal any relevant impact of tsDCS on the intracortical excitability of the somatosensory cortex by altering PSS patterns after stimulation of the TN. However, as evidence for supraspinal effects of tsDCS has been provided, further research is required to reveal the specific mechanisms of tsDCS focusing on patients’ conditions and thereby strengthening the implementation for clinical use of this promising non-invasive and low-cost technique.

## Methods

The protocol of this single-blinded, randomized and placebo-controlled crossover study was approved by the Ethics Committee of the Medical Faculty of the Ruhr-University Bochum, Germany (No. 4358-12; approved on 25 May 2012). All actions were in accordance with the Declaration of Helsinki and CONSORT guidelines were followed (Supplement 2). The trial was registered at German Clinical Trials Register (DRKS-ID: DRKS00012651; registered 04/08/2017). Written and informed consent of all participants was obtained before data acquisition.

### Participants

Fifteen healthy and right-handed young men were recruited via local advertisement and consecutively included. To eliminate sex differences only men were included. Exclusion criteria were any previous medical condition (such as obesity, diabetes, chronic pain, seizures or other neurological or psychiatric comorbidities) and any regular use of medication.

### TN SEP and paired stimulation

SEPs of the tibial nerve were acquired using a block electrode (distance between anode and cathode: 20 mm) placed over the tibial nerve stem at the right medial malleolus. To ensure a reliable supramaximal nerve stimulation, current intensity of the square wave (0.2 ms) was set approximately 2 mA above motor threshold intensity inducing a muscular twitch of the plantar muscles^38^. Table 1. A Digitimer DS7A Current Stimulator (Digitimer Ltd, Hertfordshire, UK) timed by a custom-built timing device (microcontroller board, Arduino) generated stimuli according to the following fixed scheme: a single stimulus (SS) was followed by paired stimuli with an interstimulus interval (ISI) of 60 ms (PS60), which were again followed by paired stimuli with an ISI of 90 ms (PS90). Previous studies demonstrated that an ISI of 60–90 ms leads to a reliable PSS (see below)^66^. As the recovery time of the tibial nerve after paired stimulation is around 250 ms, stimulation frequency was set to 2 Hz giving enough time to bridge the refractory phase of cortical potentials at an ISI of 90 ms^66^. SEPs were recorded by an electrode placed at Cz’ referenced to frontopolar cortex (Fp) and with the ground electrode placed above the forehead (Fpz). Electrode locations were accurately cleaned and degreased before electrode placement. Resistances were kept below 5 kOhm. For averaging, 400 repetition cycles were recorded using Brain Vision Recorder software (Version 1.02, Brain Products GmbH, Gilching, Germany). We used Brain Vision Analyzer software (Version 1.05, Brain Products GmbH, Gilching, Germany) for further offline analysis of the SEPs. Averaged peak-to-peak amplitudes and latencies (P40-N50 component^55^) of each SS SEP were manually detected and analyzed. Figure 2A. For the acquisition of PSS ratios, we analyzed peak-to-peak amplitudes evoked by the second stimulus (A2) after digital subtraction of the single stimulus (A2s) and divided it by the response of the first stimulus before linear subtraction (A1). Figure 2B,C. PSS for PS60 and PS90 was expressed as a ratio of these amplitudes (A2s/A1)^36,38,47^.

### TsDCS

TsDCS was generated by a neuroConn DC-Stimulator Plus (neuroConn GmbH, Ilmenau, Germany) and applied by a pair of rubber electrodes (5 × 7 cm; 35 cm^2^; 2 mm thick) each positioned longitudinally). Analogously to Schweizer et al.^34,67^, the spinal electrode was centered with its lower end above the spinous process of the twelfth thoracic vertebra, the reference electrode was attached above the left scapula spine with its medial edge aligned with the medial margin of the scapula. Ten20 conductive paste (Weaver and Company, Aurora, Colorado, USA) was used to minimize resistances and to contribute to an even skin contact of the electrodes. Using a maximum current of 2.5 mA at a current density of 0.071 mA/cm^2^, a total charge of 63.9 mC/cm^2^ was supplied complying to general safety criteria^68^. Stimulation duration was set to 870 s with an additional ramp up of ten seconds and a ramp down of 20 s, hence a total stimulation time of 15 min. The spinal electrode determined the polarity (cathodal/anodal). Cathodal setup was used for sham procedure with the stimulation being limited to 15 s of 1.5 mA preceded by a ramp up of 10 s and followed by a ramp down of 20 s as well as a shammed stimulation of 855 s, resulting in a cumulative pretended stimulation time of 15 min. In detail, subjects were told that the stimulation would last for 15 min.

### Experimental design

Trial execution took place in the laboratories of the BG-University Hospital Bergmannsheil in Bochum, Germany. Participants were seated comfortably and semi-prostrated with their heads slightly flexed. Lights were dimmed, acoustics were quiet and room temperature was acclimated to 22 °C. Blinded subjects underwent cathodal, anodal and sham stimulation each during an individual out of three sessions. Sessions were scheduled at least 7 days apart at an analog time of day to avoid residual and daytime effects. The sequence of tsDCS polarity was randomized computer-generated with variable block length for each subject. SEPs were recorded before (baseline), immediately (T0) as well as 30 (T30) and 60 min (T60) after tsDCS (Fig. 1).

### Statistical analysis

Statistical analysis was carried out using IBM SPSS Statistics software (Version 25; IBM Corp., Armonk, New York, USA) by a blinded statistician. To check for inconsistencies and carryover effects, baseline data for amplitudes and latencies of each single stimulation SEP were compared by calculating a one-way analysis of variance (ANOVA) with the factor polarity (three levels: cathodal, anodal, sham). Based on normal distribution (histograms; see example in Supplement 3), data of amplitudes and latencies for SS and amplitude ratios for both PS60 and PS90 were analyzed using ANOVA for repeated measurements (rmANOVA) with Greenhouse–Geisser correction to test for main effects [’polarity’ (three levels: cathodal, anodal or sham) and ‘time’ (four levels: baseline, T0, T30, T60)]. The main factors and interactions (polarity*time) were specifically of interest to analyze for temporal effects (time) due to different tsDCS (polarity) on SS and PSS. A test for polarity sequence interaction as part of the crossover design was performed. The level of significance was set to p < 0.05.

## Supplementary Information

Supplementary Information 1.

Supplementary Information 2.

## Data Availability

The datasets generated and analyzed during this study are available from the corresponding author on reasonable request.
