# Supplementary material for: Transcutaneous spinal direct current stimulation shows no effect on paired stimulation suppression of the somatosensory cortex
Source: Sci Rep. 2020 Dec 15;10:22010. doi: 10.1038/s41598-020-79131-2 (PMC7738485; doi:10.1038/s41598-020-79131-2)
Supplement: Supplementary file 1 — Supplementary Information 1. [file 41598_2020_79131_MOESM1_ESM.docx]

Trial Protocol

(German version dated: 5/2012; translated into English: 6/2020)

**Title**

Influence of transcutaneous spinal direct current stimulation (tsDCS) on paired-pulse inhibition of the somatosensory cortex after stimulation of the posterior tibial nerve (tibial nerve SEP)

**Acronym**

None

**Website**

None

**Name and address of the primary investigator**

PD Dr. Christine H. Meyer-Frießem

Department of Anesthesiology, Intensive Care and Pain Management

Formerly: Department of Anesthesiology, Intensive Care, Palliative Medicine and Pain Management

BG-Universitätsklinikum Bergmannsheil gGmbH

Bürkle-de-la-Camp-Platz 1

44789 Bochum, Germany

Mail: meyer-friessem@ains-bergmannsheil.de

Phone: +49 234 302 6826

Fax: +49 234 302 6834

**Study center**

BG-Universitätsklinikum Bergmannsheil gGmbH

Bürkle-de-la-Camp-Platz 1

44789 Bochum, Germany

**In case of multicenter trial: coordinating center**

Not applicable (monocentric, national)

**Ethics**

Ethical approval will be obtained from the Ethic Committee of the Medical Department of Ruhr University Bochum.

**Registration**

Registration will be carried out on German Clinical Trials Register (www.drks.de).

**Sponsors**

Internal sources:

Department of Anesthesiology, Intensive Care and Pain Management

Formerly: Department of Anesthesiology, Intensive Care, Palliative medicine and Pain Management

and

Department of Neurology

BG-Universitätsklinikum Bergmannsheil gGmbH

Bürkle-de-la-Camp-Platz 1

44789 Bochum, Germany

Mail: meyer-friessem@ains-bergmannsheil.de

Website: www.bergmannsheil.de

Phone: +49 234 302 6826

Fax: +49 234 302 6834

**Purpose**

Primary objective is to evaluate the neuroplastic effect of transcutaneous spinal direct current stimulation (tsDCS) on the primary sensory cortex of the brain.

**Rationale**

Transcranial direct current stimulation (tDCS) is a clinically practiced technique to modulate cortical excitability of the brain. Being cheap and non-invasive, tDCS has gained increasing relevance in the treatment of patients with strokes, seizures, chronic pain, schizophrenia or depression (Hummel et al. 2005, Fregni et al. 2006, Paulus et al. 2008). If general safety guidelines are being observed, non-reversible lesions of skin or neural tissue are almost impossible (Nitsche et al. 2008). Known as of today, current flow direction can cause specific effects. Anodal stimulation leads to an increased spontaneous neural activity of the stimulated cortex whereas cathodal stimulation has an opposite effect. Depending on current intensity, frequency and duration of the stimulation, these changes of neural resting potential can last longer than one hour (Nitsche et al. 2003, Priori et al. 2003).

A recent trend is the possibility to apply the procedure to modulate spinal neuroplasticity: transcutaneous spinal direct current stimulation (tsDCS). The effect of invasive treatments of the spinal cord like spinal cord stimulation (SCS) is already proven (Foletti et al. 2007). Analogously, non-invasive tsDCS seems to also bear a high potential. In human trials, anodal tsDCS has already been proven to alter sensory pathways offside its site of action by modulation of somatosensory evoked potentials (SEP) (Cogiamanian et al. 2008).

Our goal is to establish tsDCS in Bochum and thereby be able to investigate its potential and its mechanisms of action in the modulation of neural processing and consequently on pain perception, pain processing and pain chronification.

In a first general approach we would like to examine the central neuromodulatory capability of tsDCS by analyzing its effect on the excitability of the primary somatosensory cortex. This can be done by analyzing paired pulse suppression (PSS) patterns after stimulation of the posterior tibial nerve (tibial nerve somatosensory potentials) as PSS is an established tool to examine cortical excitability (Höffken et al. 2010). Fourteen healthy men are supposed to undergo the treatment to check for an effect. It should be distinguished if a cathodal stimulation leads to a decreased excitability and if an anodal stimulation leads to an increased excitability of the primary somatosensory cortex.

We hope to gain knowledge about sensory processing at spinal and cortical level and eventually about its impact on pain processing.

**Primary/secondary endpoints**

- Anodal tsDCS increases cortical excitability
- Cathodal tsDCS decreases cortical excitability
- tsDCS changes paired pulse suppression patterns up to 60 minutes

Primary outcome: tsDCS leads to changes in latencies and amplitudes of cortical paired-pulse tibial nerve SEP depending on the stimulation pattern (anodal, cathodal)

Secondary outcome: Changes endure up to 60 minutes after the stimulation ended

**Examined disease/health problem**

Healthy volunteers

**Concern of scientific intention**

Experimental

**Estimated overall duration of trial**

18 months

**Probands**

Estimated sample size:

16 healthy young men

Due to missing existing literature the sample size cannot be calculated. Typically, there are around twelve to fifteen subjects investigated in tsDCS trials.

A maximum of sixteen probands will be examined at three different points of time. Every examination takes between two to three hours. Each examination is scheduled at least seven days apart.

By experience from other internal projects dropout rate is expected to be between 8 to 10 %.

Planned statistics:

Data analysis will be carried our using IBM SPSS Statistics software. Data will be analyzed using repeated measurements ANOVA. Level of significance will be .05.

How long will the recruitment of probands be?

Recruitments of probands will be individual and close to scheduled measurement. Specific prearrangements are not necessary.

Countries, where probands will be recruited:

Germany

Inclusion criteria:

- Voluntariness
- Male sex
- Age between 18 and 40 years at time of measurement
- Good health (see exclusion criteria)
- Dexterity
- Sufficient knowledge of the German language
- Written informed consent

Exclusion criteria:

- Withdrawal of agreement
- Wish for termination
- Female gender
- Age < 18 or > 40 years
- Sinistrality
- Ambidexterity
- Pre-existing neurological condition
- Pre-existing psychiatric condition
- Comorbidity; use of medication
- Employees of the researching medic, or the facility which is participating in this clinical research or other studies

Insurance:

Not planned

Recompense:

10,00 €/ h; probands will receive the recompense at the end of all three measurements.

Benefits for the proband:

There is no direct benefit for the proband.

Risks for the proband:

The only known complications of tsDCS are mild itchiness and skin redness. tsDCS of 15 minutes (as planned) did not lead to an increase of NSE (neuron specific enolase) in serum as a sign of nerve damage (Cogiamanian et al. 2008). Applied correctly and in accordance with general safety criteria tDCS is of minimal risk (Nitsche et al. 2008). These safety criteria will be strictly followed.

SEPs are daily used routine diagnostics and cause no harm to the probands.

**Value for medical science**

tsDCS is a topic of current interest. If it is possible to establish tsDCS in Bochum and to achieve success in for example nociceptive neuromodulation, then this project could become the entry point to a promising research field. The non-invasive and reasonable procedure could potentially lead to benefits in the treatment of conditions like chronic pain, fibromyalgia, neuralgia, chronic regional pain syndrome or spinal injuries.

**Trial characteristics**

Type of study: interventional study

Study design: placebo-controlled, stimulation-randomized study

Blinding: single blinded

Who is blinded: subjects, additionally statistician

Control group: no control group; crossover: anodal, cathodal, placebo

Trial purpose: fundamental research

Group assignment: cross-over design

Trial phase: not applicable

Off-label drug use: not applicable

**Methodology**

A maximum of 16 trial participants will undergo temporally isolated anodal, cathodal or sham transcutaneous spinal direct current stimulations after a baseline tibial nerve SEP. Subsequently, there will be immediate tibial nerve SEP recordings as well as delayed ones after 30 and 60 minutes to capture a possible effect over time. Latencies and amplitudes as well as amplitude ratios of double pulse SEPs of the cortical P40 answer before and after tsDCS will be compared. The basis of analyzing cortical excitability will be the analysis of paired pulse inhibition ratios generated by double pulse SEPs.

Transcutaneous spinal direct current stimulation (tsDCS):

- Cathodal stimulation tsDCS, Cross-over, 15 minutes over T10-12, 2.5 mA

- Anodal stimulation tsDCS, Cross-over, 15 minutes over T10-12, 2.5 mA

- Sham tsDCS, Cross-over, 15 minutes over T10-12

Stimulation will be generated by a neuroConn DC-Stimulator Plus (neuroConn GmbH, Ilmenau, Germany) and applied by a pair of rubber electrodes (5 x 7 cm; 35 cm^2^; each positioned longitudinally). The spinal electrode will be centered above the spinous process of the twelfth thoracic vertebra, the second electrode will be attached above the left scapula spine. Ten20 conductive paste (Weaver and Company, Aurora, Colorado, USA) will be used for skin contact. Stimulation duration will be set to 870 s with an additional fade-in of 10 s and a ramp-out of 20 s. The spinal electrode will determine the polarity (cathodal/anodal). Cathodal setup will be used for sham stimulation limited to 15 s of 1.5 mA (plus a fade-in of 10 s and a ramp-down of 20 s).

Somatosensory evoked potentials (SEP):

SEP will be conducted in accordance to internal clinical standards. Tibial nerve SEPs will be acquired using a stimulation block placed over the tibial nerve stem posterodistal to the right medial malleolus. Current intensity will be set approximately 2 mA above motor threshold intensity inducing a muscular twitch of the plantar muscles. A Digitimer DS7A Current Stimulator (Digitimer Ltd, Hertfordshire, UK) timed by a timing device (custom-built Arduino microcontroller board) will generate the pulses according to the following scheme: a single pulse (SP) will be followed by a double pulse (DP) with an interstimulus interval (ISI) of 60 ms (DP60), which will again be followed by a DP with an ISI of 90 ms (DP90). Repetition rate will be set to 2 Hz to give enough time to bridge the refractory phase. SEPs will be recorded by an electrode placed at Cz’ referenced to frontopolar cortex (Fp). The ground electrode will be placed above the forehead (Fpz). Electrode locations must be accurately cleaned and degreased before electrode placement. Resistances are supposed to be kept below 5 kOhm, better be 0 to 2 kOhm. For averaging, 400 repetition cycles will be recorded using Brain Vision Recorder software (Brain Products GmbH, Gilching, Germany). For analysis Brain Vision Analyzer software (Brain Products GmbH, Gilching, Germany) will be used. Averaged peak-to-peak amplitudes and latencies of the cortical P40 complex will be detected and analyzed. For the acquisition of double pulse inhibition ratios, we will analyze peak-to-peak amplitudes evoked by the second pulse after digital subtraction of the single pulse and then refer it to the response of the first pulse before linear subtraction. Double pulse inhibition will be expressed as a ratio of these amplitudes.

Set up:

Participants will be seated comfortably and semi-prostrated with their heads slightly flexed. Lights will be dimmed, acoustics will be quiet. Subjects will be told to close their eyes and relax. Subjects will undergo cathodal, anodal and sham stimulation each during an individual out of three sessions, scheduled with an interval of at least seven days from each other. The sequence of tsDCS polarity will be randomized computer-generated (variable block length) for each subject. SEPs will be recorded before (baseline), immediately (T0) as well as 30 (T30) and 60 minutes (T60) after tsDCS.

**Information on medical device**

Name: neuroConn DC-stimulator Plus

Manufacturer: neuroConn GmbH, Albert-Einstein-Straße 3, 8693 Ilmenau, Germany

CE-Sign: yes

Product brochure existing: yes

Eligible for medical indication: yes

**Study personnel**

Christine H. Meyer-Frießem: Principle investigator; responsible for all issues

and head of organization

Jan H. Bettmann: Investigator, data collection and management

Lauren M. Schweizer (formerly Haag): Investigator; statistics

Peter K. Zahn: Supervisor

Martin Tegenthoff: Supervisor

Oliver Höffken: Supervisor
